# Supplementary material for: Improving Turnaround Times for Routine Antimicrobial Sensitivity Testing Following European Committee on Antimicrobial Susceptibility Testing Methodology in Patients with Bacteraemia
Source: Antibiotics (Basel). 2024 Nov 17;13(11):1094. doi: 10.3390/antibiotics13111094 (PMC11591232; doi:10.3390/antibiotics13111094)
Supplement: Supplementary file 1 [file antibiotics-13-01094-s001.zip › antibiotics-3236245-supplementary.pdf]

Supplementary Materials

Figure S1. Summary of Staphylococcus organism samples, with MALDI-TOF MS organism identification from six and 24-hour culture plates.

		Cefoxitin				Erythromycin				Clindamycin				Co-trimoxazole				Tetracycline				Chloramphenicol				Gentamicin				Linezolid				Rifampicin				Fusidic Acid				Ciprofloxacin				Mupirocin			
Reference	Organism identification	6 h		24 h		6 h		24 h		6 h		24 h		6 h		24 h		6 h		24 h		6 h		24 h		6 h		24 h		6 h		24 h		6 h		24 h		6 h		24 h		6 h		24 h		6 h		24 h	
Staph 1	*Staphylococcus aureus*	29	S	29	S	28	S	28	S	28	S	28	S	31	S	32	S	26	S	26	S	28	S	27	S	22	S	22	S	26	S	27	S	34	S	32	S	32	S	30	S	24	S	23	S	36	S	34	S
Staph 2	*Staphylococcus aureus*	27	S	28	S	26	S	26	S	24	S	24	S	32	S	32	S	26	S	26	S	25	S	26	S	20	S	20	S	26	S	26	S	28	S	28	S	28	S	28	S	22	S	22	S	35	S	35	S
Staph 3	*Staphylococcus aureus*	27	S	28	S	25	S	24	S	24	S	23	S	30	S	30	S	24	S	24	S	22	S	21	S	20	S	20	S	30	S	23	S	29	S	29	S	28	S	24	S	28	S	27	S	31	S	30	S
Staph 4	*Staphylococcus aureus*	29	S	28	S	28	S	28	S	25	S	26	S	32	S	32	S	32	S	30	S	27	S	27	S	22	S	22	S	30	S	30	S	36	S	34	S	26	S	29	S	24	S	24	S	33	S	35	S
Staph 5	*Staphylococcus aureus*	29	S	28	S	26	S	27	S	27	S	27	S	30	S	31	S	28	S	27	S	26	S	26	S	21	S	22	S	28	S	26	S	32	S	31	S	34	S	31	S	25	S	24	S	34	S	35	S
Staph 6	*Staphylococcus aureus*	30	S	38	S	29	S	28	S	28	S	28	S	32	S	33	S	29	S	28	S	25	S	25	S	21	S	22	S	26	S	26	S	32	S	32	S	32	S	31	S	23	S	24	S	38	S	36	S
Staph 7	*Staphylococcus aureus*	27	S	26	S	26	S	26	S	24	S	25	S	27	S	28	S	24	S	26	S	23	S	24	S	20	S	20	S	25	S	25	S	28	S	29	S	27	S	28	S	21	S	22	S	30	S	32	S
Staph 8	*Staphylococcus aureus*	25	S	26	S	6	R	6	R	D	R	D	R	30	S	31	S	26	S	26	S	24	S	24	S	22	S	22	S	25	S	26	S	31	S	33	S	10	R	11	R	25	S	24	S	31	S	32	S
Staph 9	*Staphylococcus aureus*	26	S	27	S	6	R	6	R	D	R	D	R	29	S	30	S	25	S	24	S	22	S	22	S	21	S	22	S	25	S	24	S	30	S	29	S	10	R	11	R	23	S	23	S	29	I	29	I
Staph 10	*Staphylococcus aureus*	27	S	26	S	27	S	27	S	26	S	26	S	27	S	30	S	26	S	27	S	23	S	23	S	22	S	23	S	25	S	27	S	30	S	33	S	9	R	11	R	26	S	25	S	33	S	33	S
MRSA ATCC33591	**Staphylococcus aureus*	6	R	6	R	6	R	6	R	6	R	6	R	24	S	24	S	6	R	6	R	10	R	10	R	16	R	16	R	23	S	27	S	30	S	28	S	26	S	25	S	21	S	24	S	30	S	32	S
Staph 11	*Staphylococcus epidermis*	15	R	15	R	6	R	6	R	6	R	6	R	13	R	12	R	15	R	15	R	25	S	26	S	11	R	13	R	30	S	31	S	40	S	42	S	9	R	8	R	15	R	15	R				
Staph 12	*Staphylococcus epidermis*	38	S	31	S	31	S	29	S	30	S	29	S	32	S	32	S	30	S	29	S	24	S	25	S	25	S	24	S	26	S	27	S	46	S	46	S	9	R	10	R	36	S	34	S				
Staph 13	*Staphylococcus epidermis*	15	R	14	R	6	R	6	R	17	R	19	R	9	R	6	R	10	R	11	R	25	S	29	S	8	R	6	R	26	S	32	S	36	S	40	S	12	R	13	R	18	R	16	R				
Staph 14	*Staphylococcus epidermis*	10	R	6	R	31	S	36	S	28	S	32	S	6	R	6	R	24	S	28	S	26	S	28	S	10	R	9	R	28	S	30	S	36	S	38	S	29	S	30	S	6	R	6	R				
Staph 15	*Staphylococcus epidermis*	21	R	23	R	30	S	30	S	27	S	28	S	14	I	16	I	23	S	26	S	23	S	28	S	7	R	6	R	26	S	30	S	35	S	42	S	27	S	28	S	30	S	34	S				
Staph 16	*Staphylococcus epidermis*	33	S	35	S	8	R	8	R	28	S	30	S	26	S	30	S	22	S	24	S	25	S	30	S	20	R	20	R	26	S	26	S	41	S	42	S	10	R	14	R	27	S	30	S				
Staph 17	*Staphylococcus capitis*	6	R	6	R	31	S	33	S	34	S	30	S	35	S	36	S	28	S	24	S	26	S	29	S	10	R	6	R	29	S	32	S	36	S	38	S	8	R	9	R	32	S	32	S				
Staph 18	*Staphylococcus haemolyticus*	6	R	6	R	8	R	6	R	13	R	11	R	6	R	6	R	24	S	23	S	26	S	25	S	6	R	6	R	26	S	26	S	6	R	6	R	29	S	30	S	6	R	6	R				
Staph 19	*Staphylococcus hominis*	18	R	15	R	6	R	6	R	28	S	34	S	6	R	6	R	6	R	6	R	28	S	33	S	26	S	26	S	28	S	30	S	38	S	42	S	9	R	14	R	30	S	32	S				
Staph 20	*Staphylococcus hominis*	16	R	17	R	6	R	6	R	D	R	D	R	6	R	6	R	6	R	6	R	26	S	26	S	25	S	26	S	25	S	26	S	36	S	36	S	10	R	11	R	26	S	28	S				
Staph 21	*Staphylococcus hominis*	14	R	15	R	6	R	6	R	D	R	D	R	6	R	6	R	29	S	31	S	25	S	28	S	18	R	19	R	28	S	30	S	42	S	42	S	9	R	10	R	30	S	32	S				

Antibiotic disc zone sizes in mm are recorded for each antibiotic from six hour and 24-hour culture plates set up for AST. The organism is then recorded as sensitive (S), resistant (R), susceptible with increased exposure (I), or in an area of technical uncertainty (ATU), based on disc zone sizes according to EUCAST methodology. Control organism is signified with a '*'.

Figure S2. Summary of Enterobacterales organism samples, with MALDI-TOF MS organism identification from six and 24-hour culture plates.

		Ampicillin				Amox-clav				Ertapenem				Cefuroxime				Cefotaxime				Co trimoxazole				Ciprofloxacin				Gentamicin				Meropenem				Ceftazidime				Amikacin				Pip-taz			
Reference	Organism identification	6h		24h		6h		24h		6h		24h		6h		24h		6h		24h		6h		24h		6h		24h		6h		24h		6h		24h		6h		24h		6h		24h		6h		24h	
Coli 1	*Enterobacter cloacae*	6	R	6	R	6	R	6	R	31	S	31	S	19	S	19	S	24	S	24	S	23	S	23	S	30	S	31	S	18	S	19	S	31	S	31	S	24	S	25	S	19	S	19	S	22	S	22	S
Coli 2	*Escherichia coli*	21	S	18	S	24	S	21	S	37	S	35	S	22	S	21	S	29	S	28	S	30	S	28	S	33	S	33	S	20	S	20	S	34	S	35	S	27	S	26	S	20	S	20	S	25	S	24	S
Coli 3	*Escherichia coli*	6	R	6	R	6	R	6	R	34	S	35	S	22	S	24	S	30	S	30	S	26	S	27	S	29	S	30	S	19	S	19	S	31	S	32	S	28	S	29	S	20	S	20	S	20	S	21	S
Coli 4	*Escherichia coli*	6	R	6	R	10	R	10	R	31	S	35	S	18	R	18	R	23	S	24	S	26	S	27	S	31	S	32	S	18	S	18	S	30	S	32	S	23	S	24	S	18	S	19	S	20	S	22	S
Coli 5	*Escherichia coli*	6	R	6	R	10	R	10	R	32	S	33	S	19	S	19	S	23	S	24	S	27	S	27	S	32	S	32	S	18	S	18	S	31	S	32	S	24	S	24	S	18	S	19	S	21	S	21	S
Coli 6	*Escherichia coli*	18	S	17	S	21	S	21	S	32	S	33	S	22	S	23	S	28	S	28	S	6	R	6	R	6	R	6	R	18	S	18	S	32	S	33	S	26	S	26	S	18	S	18	S	24	S	25	S
Coli 7	*Escherichia coli*	6	R	6	R	17	R	17	R	31	S	32	S	21	S	20	S	27	S	27	S	6	R	6	R	22	ATU	22	ATU	18	S	19	S	31	S	32	S	26	S	27	S	18	S	18	S	23	S	23	S
Coli 8	*Escherichia coli*	6	R	6	R	9	R	9	R	32	S	34	S	19	S	19	S	23	S	23	S	26	S	28	S	32	S	33	S	18	S	18	S	31	S	31	S	24	S	24	S	19	S	19	S	21	S	21	S
Coli 9	*Escherichia coli*	6	R	6	R	18	R	18	R	30	S	31	S	24	S	24	S	28	S	28	S	6	R	6	R	32	S	33	S	18	S	19	S	31	S	31	S	26	S	27	S	18	S	19	S	23	S	25	S
Coli 10	*Escherichia coli*	6	R	6	R	7	R	7	R	29	S	29	S	12	R	13	R	9	R	9	R	6	R	6	R	12	R	12	R	19	S	20	S	31	S	34	S	14	R	14	R	19	S	20	S	19	ATU	19	ATU
Coli 11	*Escherichia coli*	19	S	19	S	21	S	22	S	33	S	33	S	22	S	23	S	26	S	29	S	14	S	12	I	25	S	28	S	19	S	20	S	33	S	35	S	24	S	30	S	19	S	20	S	24	S	25	S
IMP NCTC13476	**Escherichia coli*	6	R	6	R	13	R	14	R	16	R	17	R	6	R	6	R	6	R	6	R	6	R	6	R	6	R	6	R	6	R	6	R	21	I	21	I	6	R	6	R	19	S	18	S	18	R	18	R
Coli 12	*Klebsiella aerogenes*	6	R	6	R	8	R	6	R	29	S	30	S	22	S	22	S	25	S	25	S	25	S	24	S	30	S	32	S	20	S	20	S	30	S	30	S	24	S	24	S	21	S	21	S	21	S	21	S
Coli 13	*Klebsiella pneumoniae*	6	R	6	R	17	R	18	R	28	S	27	S	6	R	6	R	6	R	6	R	6	R	6	R	22	ATU	22	ATU	19	S	18	S	31	S	29	S	11	R	11	R	20	S	21	S	22	S	21	S
Coli 14	*Klebsiella pneumoniae*	6	R	6	R	23	S	22	S	31	S	31	S	11	R	10	R	27	S	26	S	26	S	24	S	27	S	27	S	19	S	19	S	32	S	30	S	25	S	26	S	22	S	21	S	22	S	21	S
Coli 15	*Klebsiella pneumoniae*	6	R	6	R	23	S	22	S	30	S	32	S	23	S	23	S	28	S	28	S	6	R	6	R	22	ATU	22	ATU	19	S	19	S	31	S	31	S	26	S	26	S	20	S	20	S	21	S	21	S
Coli 16	*Klebsiella pneumoniae*	6	R	6	R	22	S	22	S	31	S	30	S	23	S	22	S	27	S	27	S	6	R	6	R	21	R	21	R	19	S	19	S	30	S	30	S	26	S	25	S	20	S	20	S	20	S	20	S
Coli 17	*Klebsiella pneumoniae*	6	R	6	R	21	S	23	S	29	S	30	S	22	S	23	S	26	S	28	S	25	S	25	S	27	S	28	S	19	S	19	S	30	S	29	S	25	S	26	S	19	S	20	S	20	S	20	S
Coli 18	*Klebsiella pneumoniae*	6	R	6	R	23	S	25	S	29	S	31	S	23	S	24	S	28	S	28	S	27	S	28	S	29	S	29	S	19	S	20	S	29	S	31	S	25	S	26	S	20	S	20	S	21	S	22	S
ESBL ATCC700603	**Klebsiella pneumoniae*	6	R	6	R	18	R	17	R	28	S	28	S	13	R	12	R	15	R	14	R	16	S	19	S	23	ATU	24	ATU	12	R	12	R	30	S	31	S	11	R	10	R	19	S	21	S	18	R	17	R
VIM1 NCTC13439	**Klebsiella pneumoniae*	6	R	6	R	10	R	6	R	20	R	20	R	6	R	6	R	6	R	6	R	6	R	6	R	10	R	10	R	18	S	19	S	24	S	23	S	6	R	6	R	20	S	20	S	11	R	11	R
BAA ATCCBAA2814	**Klebsiella pneumoniae*	6	R	6	R	6	R	6	R	6	R	6	R	6	R	6	R	6	R	6	R	6	R	6	R	6	R	6	R	16	R	16	R	6	R	6	R	6	R	6	R	9	R	9	R	6	R	6	R
KPC NCTC13438	**Klebsiella pneumoniae*	6	R	6	R	6	R	6	R	6	R	6	R	6	R	6	R	6	R	6	R	6	R	6	R	6	R	6	R	16	R	16	R	6	R	7	R	6	R	6	R	10	R	11	R	6	R	6	R
OXA48 NCTC13442	**Klebsiella pneumoniae*	6	R	6	R	6	R	6	R	17	R	17	R	17	R	17	R	19	I	18	I	6	R	6	R	15	R	15	R	19	S	21	S	23	S	22	S	23	S	22	S	21	S	21	S	7	R	6	R
NDM1 NCTC13443	**Klebsiella pneumoniae*	6	R	6	R	6	R	6	R	6	R	6	R	6	R	6	R	6	R	6	R	6	R	6	R	6	R	6	R	6	R	6	R	6	R	6	R	6	R	6	R	6	R	6	R	6	R	6	R
Coli 19	*Proteus mirabilis*	6	R	6	R	22	S	22	S	30	S	31	S	22	S	22	S	30	S	31	S	6	R	6	R	24	ATU	24	ATU	18	S	18	S	29	S	30	S	30	S	30	S	16	R	16	R	27	S	28	S
Coli 20	*Serratia marcescens*	6	R	6	R	6	R	6	R	32	S	33	S	6	R	6	R	25	S	29	S	30	S	30	S	32	S	37	S	17	S	19	S	30	S	33	S	28	S	29	S	18	S	20	S	24	S	27	S

Control organisms are signified with a '*'.

Figure S3. Summary of Enterococcus/Streptococcus organism samples, with MALDI-TOF MS organism identification from six and 24-hour culture plates.

		Ampicillin				Linezolid				Teicoplanin				Vancomycin			
Reference	Organism identification	6h		24h		6h		24h		6h		24h		6h		24h	
Ent 1	*Enterococcus faecalis*	19	S	18	S	23	S	23	S	18	S	18	S	15	S	15	S
Ent 2	*Enterococcus faecalis*	18	S	15	S	23	S	21	S	17	S	16	S	14	S	15	S
Ent 3	*Enterococcus faecalis*	18	S	19	S	21	S	22	S	16	S	17	S	13	S	13	S
Ent 4	*Enterococcus faecium*	6	R	6	R	24	S	24	S	17	S	25	S	-	-	22	S
Ent 5	*Enterococcus faecium*	6	R	6	R	30	S	38	S	20	S	22	S	20	S	21	S
Ent 6	*Enterococcus faecium*	6	R	6	R	26	S	23	S	19	S	19	S	17	S	18	S
Ent 7	*Enterococcus faecium*	6	R	6	R	23	S	23	S	18	S	18	S	18	S	18	S
Ent 8	*Enterococcus faecium*	6	R	6	R	24	S	22	S	6	R	6	R	6	R	6	R
Ent 9	*Enterococcus faecium*	6	R	6	R	23	S	23	S	8	R	8	R	6	R	6	R
VRE NCTC12202	**Enterococcus faecium*	6	R	6	R	22	S	22	S	6	R	6	R	6	R	6	R

		Linezolid				Teicoplanin				Vancomycin				Erythromycin				Tetracycline				Chloramphenicol				Rifampicin				Co trimoxazole				Penicillin				Clindamycin			
Reference	Organism Identification	6h		24h		6h		24h		6h		24h		6h		24h		6h		24h		6h		24h		6h		24h		6h		24h		6h		24h		6h		24h	
Strep 1	*Streptococcus algalactiae*	21	S	24	S	15	S	15	S	15	S	16	S	23	S	26	S	12	R	14	R	23	S	27	S	24	S	26	S	27	S	29	S	22	S	22	S	20	S	22	S
Strep 2	*Streptococcus algalactiae*	23	S	23	S	16	S	17	S	15	S	17	S	25	S	27	S	29	S	29	S	24	S	28	S	26	S	30	S	26	S	29	S	23	S	23	S	22	S	25	S
Strep 3	*Streptococcus algalactiae*	21	S	24	S	16	S	17	S	16	S	17	S	6	R	6	R	11	R	13	R	24	S	27	S	24	S	27	S	27	S	30	S	21	S	24	S	6	R	11	R
Strep 4	*Streptococcus algalactiae*	21	S	25	S	15	S	17	S	16	S	17	S	6	R	6	R	11	R	12	R	22	S	26	S	23	S	27	S	25	S	30	S	21	S	23	S	6	R	10	R
Strep 5	*Streptococcus algalactiae*	21	S	23	S	15	S	17	S	15	S	17	S	22	S	26	S	10	R	12	R	23	S	26	S	23	S	26	S	25	S	28	S	20	S	23	S	19	S	22	S
Strep 6	*Streptococcus dysgalactiae*	20	S	23	S	15	S	16	S	15	S	16	S	22	S	24	S	11	R	12	R	21	S	25	S	23	S	27	S	24	S	26	S	23	S	25	S	19	S	21	S
Strep 7	*Streptococcus dysgalactiae*	20	S	22	S	15	S	16	S	14	S	15	S	21	S	24	S	24	S	28	S	21	S	25	S	24	S	26	S	23	S	26	S	22	S	26	S	19	S	21	S
Strep 8	*Streptococcus dysgalactiae*	21	S	23	S	16	S	18	S	16	S	17	S	22	S	25	S	23	S	26	S	23	S	27	S	28	S	30	S	27	S	30	S	26	S	29	S	18	S	21	S
Strep 9	*Streptococcus dysgalactiae*	21	S	23	S	16	S	18	S	17	S	18	S	23	S	26	S	25	S	29	S	24	S	26	S	25	S	26	S	26	S	29	S	24	S	26	S	18	S	20	S
Strep 10	*Streptococcus pyogenes*	21	S	23	S	16	S	17	S	17	S	18	S	22	S	26	S	24	S	28	S	23	S	25	S	25	S	30	S	22	S	24	S	23	S	27	S	19	S	22	S

		Linezolid				Teicoplanin				Vancomycin				Optochin				Erythromycin				Oxacillin				Tetracycline				Chloramphenicol				Norfloxacin				Rifampicin				Co trimoxazole			
Reference	Organism Identification	6h		24h		6h		24h		6h		24h		6h		24h		6h		24h		6h		24h		6h		24h		6h		24h		6h		24h		6h		24h		6h		24h	
Strep 11	*Streptococcus pneumoniae*	22	S	22	S	17	S	17	S	17	S	17	S	13	R	13	R	23	S	24	S	24	S	24	S	27	S	28	S	24	S	25	S	16	S	16	S	26	S	27	S	23	S	24	S
Strep 12	*Streptococcus pneumoniae*	25	S	25	S	16	R	19	S	15	R	17	S	14	S	15	S	24	S	26	S	27	S	27	S	29	S	30	S	26	S	25	S	17	S	18	S	26	S	28	S	21	S	24	S

Control organism is signified with a '*'. No result available is signified with a '-'.

Figure S4. Summary of pneumonia causing organism samples, with MALDI-TOF MS organism identification from six and 24-hour culture plates.

		Ampicillin				Amox-clav				Cefotaxime				Chloramphenicol				Tetracycline				Ciprofloxacin			
Reference	Organism Identification	6h		24h		6h		24h		6h		24h		6h		24h		6h		24h		6h		24h	
Flu 1	*Haemophilus influenzae*	19	S	20	S	18	S	19	S	31	S	32	S	32	S	33	S	30	S	31	S	35	S	36	S
Flu 2	*Haemophilus influenzae*	19	S	20	S	17	S	17	S	30	S	30	S	31	S	31	S	30	S	31	S	33	S	33	S
Flu 3	*Haemophilus influenzae*	22	S	22	S	20	S	20	S	35	S	35	S	34	S	34	S	32	S	32	S	36	S	37	S
Flu 4	*Haemophilus influenzae*	19	S	21	S	17	S	19	S	31	S	32	S	33	S	33	S	32	S	33	S	36	S	37	S
Flu 5	*Haemophilus influenzae*	16	R	16	R	9	R	9	R	31	S	30	S	34	S	34	S	32	S	33	S	37	S	37	S
Flu 6	*Haemophilus influenzae*	6	R	6	R	17	S	17	S	31	S	31	S	32	S	33	S	31	S	32	S	35	S	35	S
Flu 7	*Haemophilus influenzae*	20	S	22	S	18	S	20	S	30	S	32	S	33	S	36	S	31	S	33	S	36	S	36	S
Flu 8	*Haemophilus influenzae*	23	S	23	S	21	S	22	S	32	S	33	S	33	S	34	S	32	S	33	S	36	S	36	S
Flu 9	*Haemophilus influenzae*	22	S	20	S	20	S	18	S	30	S	29	S	30	S	30	S	30	S	30	S	36	S	35	S
Flu 10	*Haemophilus influenzae*	21	S	22	S	20	S	21	S	31	S	33	S	34	S	35	S	32	S	33	S	35	S	36	S
Flu 11	*Haemophilus influenzae*	21	S	21	S	19	S	19	S	30	S	30	S	34	S	34	S	30	S	30	S	35	S	34	S
Flu 12	*Haemophilus influenzae*	20	S	21	S	18	S	20	S	31	S	34	S	31	S	33	S	31	S	33	S	33	S	35	S
Flu 13	*Haemophilus influenzae*	19	S	18	S	15	S	15	S	31	S	30	S	33	S	33	S	33	S	31	S	37	S	37	S
Flu 14	*Haemophilus influenzae*	20	S	20	S	17	S	18	S	32	S	33	S	32	S	33	S	31	S	33	S	37	S	37	S
Flu 15	*Haemophilus influenzae*	20	S	21	S	17	S	18	S	30	S	31	S	33	S	34	S	31	S	31	S	34	S	36	S
Flu 16	*Haemophilus influenzae*	17	R	17	R	16	S	16	S	29	S	28	S	33	S	33	S	31	S	31	S	36	S	37	S
Flu 17	*Haemophilus influenzae*	21	S	23	S	19	S	22	S	29	S	33	S	32	S	34	S	30	S	32	S	36	S	37	S
Flu 19	*Haemophilus influenzae*	18	S	18	S	16	S	16	S	31	S	31	S	33	S	33	S	32	S	31	S	38	S	38	S
Flu 20	*Haemophilus influenzae*	14	R	16	R	10	R	11	R	24	ATU	26	ATU	31	S	32	S	29	S	31	S	34	S	38	S
Flu 21	*Moraxella catarrhalis*	10	S	11	S	23	S	23	S	22	S	22	S	36	S	36	S	36	S	35	S	38	S	39	S
